# Anti-inflammatory and -apoptotic effects of a long-term herbal extract treatment on DSS-induced colitis in mice fed with high AGEs-fat diet

**DOI:** 10.1186/s12986-021-00603-x

**Published:** 2021-08-11

**Authors:** Fatemeh Azizian-Farsani, Marcin Osuchowski, Navid Abedpoor, Farzad Seyed Forootan, Maryam Derakhshan, Mohammad Hossein Nasr-Esfahani, Mohammad Hasan Sheikhha, Kamran Ghaedi

**Affiliations:** 1 Department of Medical Genetics, Shahid Sadoughi University of Medical Sciences, Yazd, Iran; 2 Ludwig Boltzmann Institute for Clinical and Experimental Traumatology in AUVA Research Center, Vienna, Austria; 3 Department of Cellular Biotechnology, Cell Science Research Center, Royan Institute for Biotechnology, ACECR, Royan, Salman Streets, 816513-1378 Isfahan, Iran; 4 Legal Medicine research Center, Legal Medicine Organization, Tehran, Iran; 5 Department of Pathology, Isfahan University of Medical Sciences, Isfahan, Iran; 6 Biotechnology Research Center, International Campus, Shahid Sadoughi University of Medical Sciences, Yazd, Iran; 7 Department of Cell and Molecular Biology and Microbiology, Faculty of Biological Science and Technology, University of Isfahan, Hezar Jerib Ave., Azadi Sq., 81746-73441 Isfahan, Iran

**Keywords:** Colitis, High AGEs-fat diet, Herbal extract

## Abstract

**Background:**

Obesity is associated with many comorbidities including inflammatory bowel disease (IBD). We investigated prophylactic effects of an herbal extract (HE) on the DSS-induced colitis mice challenged with high AGEs-fat diet 60% (HFD).

**Methods:**

Six-week-old C57BL/6 male mice were fed with either HFD (8 groups, 6 mice in each group), or normal diet (ND) (8 groups, 6 mice in each group). After 6 weeks, animals received HE (combination of turmeric, ginger, boswellia and cat’s claw extract) for 7 weeks in three doses (high dose (0.6 mg/g); low dose (0.15 mg/g) and mid dose (0.3 mg/g)). Next, mice were subjected to 2.5% DSS in drinking water. Control mice received ND and instead of HE and DSS they received distilled water. Obesity index markers were determined, H&E staining and TUNEL assay evaluated apoptosis. Colonic expressions of *IL-6*, *RAGE*, *AGER1*, *Sirt1*, *Bax, Bcl2, ZO-1* and *P53* were determined.

**Results:**

HE ameliorated colitis in HFD mice by reducing colonic myeloperoxidase activity (by 2.3-fold), macrophage accumulation (by 2.6-fold) and mRNA expression of *IL-6* (by 2.3-fold) in HFD mice. Moreover, HE restored *ZO-1* (by 2.7-fold), prevented apoptosis and maintained immune homeostasis. HE reduced activation of NF-κB protein (by 1.3-fold) through decreasing *RAGE* (by 1.93-fold) and up-regulation of *Sirt1* (by 7.71-fold) and prevented down-regulation of *DDOST* (by 6.6-fold) in HFD mice.

**Conclusions:**

HE ameliorated colitis in prophylactic in HFD mice and it was, at least partly, due to the restoration of the gut integrity, suppression of inflammation and apoptosis via modulation of colonic *Sirt1*, *RAGE* and *DDOST* signaling.

**Graphic abstract:**

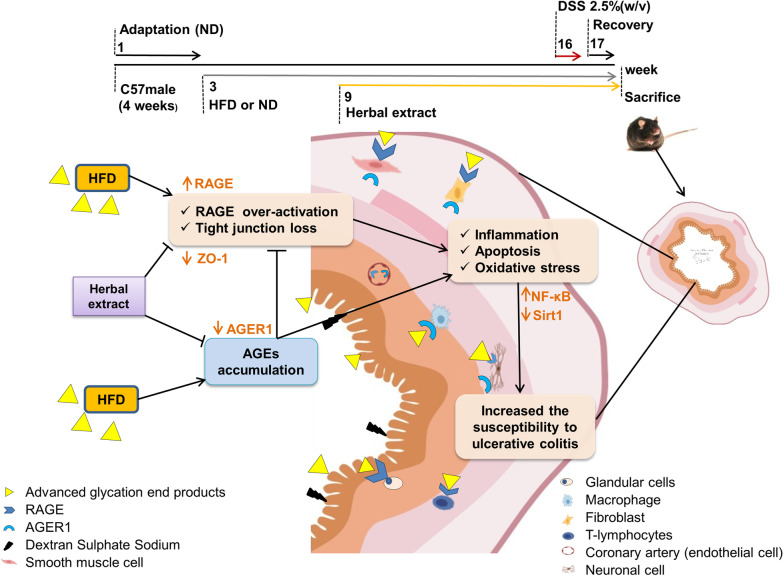

**Supplementary Information:**

The online version contains supplementary material available at 10.1186/s12986-021-00603-x.

## Background

Obesity is a rapidly growing health problem worldwide; in the US it affects 93.3 million adults [1]. Almost half of the children under 5 who were overweight or obese in 2019 lived in Asia. The rate of obesity in the European countries tripled since the 1980s [2]. Obesity prevalence is strongly correlated with the increasing frequency of inflammatory bowel disease (IBD) includes Crohn’s disease (CD) and ulcerative colitis (UC). IBD is a chronic relapsing disorder of the gut presenting with intestinal damage of varying grade. Westernized diet and life-style contribute to increased susceptibility of IBD [3]. Western diet is high in fat and advanced glycation end products (AGEs) and low in fiber. Such a composition, among other things, detrimentally modulates intestinal microbiota, enhances neutrophil migration across intestinal epithelium [4] and aggravates dextran sodium sulfate (DSS)-induced colitis [5]. Persisting high fat diet consumption induces protracted oxidative stress and NF-κB activation leading to stimulation of inflammatory response of human innate immune cells responsible for UC development [6–8].

Natural herbal substances, due to low costs and fewer adverse effects, have been widely investigated as remedies to IBD. Studies testing turmeric, ginger, boswellia and cat’s claw showed protection against IBD and colorectal cancer [9–12]. Active substances of these herbs possess multifaceted anti-inflammatory, immune-modulatory and anti-apoptotic properties. They also exhibit AGE-trapping capacity thereby boosting short chain fatty acids (SCFA)-producing intestinal microbiota and promoting epithelial barrier integrity [13–16].

Colitis is associated with an activation of receptor of advanced glycation end products (RAGE) and nuclear transcription factor kappa B (NF-κB) [17–20]. Moreover, Advanced glycation product receptor 1 (*AGER1* or dolichyl-diphosphooligosaccharide-protein glycosyltransferase subunit, *DDOST*) beneficially contributes to clearance of AGEs from circulation [21–24]. Furthermore, Colitis was shown to be associated with a decrease in silent mating type information regulation-1 (*Sirt1*) and concomitantly with NF-κB. AGEs-RAGE interaction typically decreases *Sirt1* in mesangial cells [7, 25]. Since IBD is characterized by diffuse mucosal inflammation and cell apoptosis in both CD and UC, it has been speculated that AGEs in high fat diet modulates apoptosis [26, 27]. Moreover, the effect of AGEs on promoting apoptosis and tight junctions loss was confirmed by changes in the apoptosis-associated proteins (*Bax*, *Bcl-2*) [28].

The aim of this study was two-fold. First, we investigated the hypothesized anti-obesity and attenuating effects of a *Curcuma longa*, *Boswellia Serrata*, *Gingiber officinale* and *Uncaria tomentosa* (Cat’s claw) herbal extracts (HE) mixture on DSS-induced colitis in high AGEs-fat diet male C57/BL6 mice. Second, we characterized the associated molecular signaling pathways potentially responsible for the observed effects.

## Methods

### Preparation of herbal extract

The four HEs compounds were purchased dried from Goldaru Pharmaceutical Company (Esfahan, Iran) and manufactured under GMP regulations. 500 mg of HE powder contained Turmeric (155 mg), Cat’s claw (49 mg), Ginger (148 mg) and boswellia (148 mg). HE was suspended in 45 mL sterile distilled water in a ratio of 1:90 w/v, and stirred for 2 h. Next, three different doses were made: 0.6 (High dose: HD), 0.3 (Mid dose: MD) and 0.15 mg/g (Low dose: LD). Quality control and standardization were performed and HE content was assessed by gas chromatography-mass spectrometry (GC–MS).

### Gas chromatography-mass spectrometry (GC–MS) of HE

GC–MS of HE was performed with a GC 7890 equipped with a MS 5975C detector and HP-5 ms capillary column (30 m × 0.25 mm × 0.25 µm; Agilent Co., USA). Initial column temperature was set at 40–80 °C for 3 min and retained at 295 °C for 10 min, with a heating rate of 10 °C per min. Interpretation of data on mass spectrum GC–MS was performed using National Institute of Standards and Technology (NIST) database. Spectrum of the unknown component was compared with spectrum of the known components stored in the NIST library. The name, molecular formula, nature of some components of HE and their biological activity were defined [29].

### Quality control of HE

To confirm HE stability, HE contents were measured before and after the end of study by UV-3300 spectrophotometer for curcumin as the most prominent and effective content of HE, and GC–MS for the rest of components. HE (5.0 g) was precisely weighed and dissolved in methanol and volume was adjusted to 100 mL exactly. Then 10 mL of prepared sample was diluted with ethanol 200 × and absorbance of the sample was detected by a UV-3300 spectrophotometer (Thermo, USA), at 425 nm. The amount of curcumin was calculated as follows:

$$Curcumin\%=\frac{\mathrm{A}\times {10}^{7}}{1560\times \mathrm{w }\left(\mathrm{mg}\right)}$$

A = sample absorbance at 425 nm, W = sample weight.

### Experimental design and sampling

**Fig. 1:**
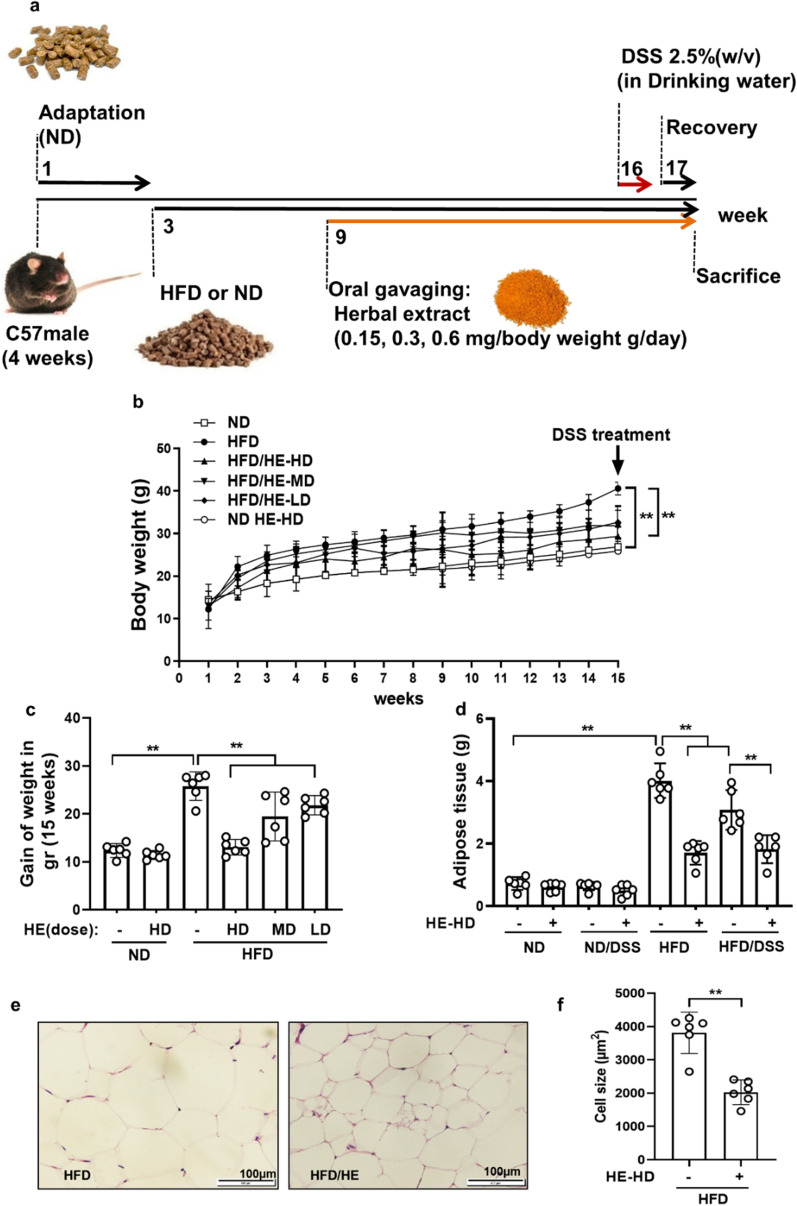
Experimental design, body, and adipose tissue weight. **a** Mice in two main groups after 2 weeks of adaptation with ND, received HFD or continued with ND for 15 weeks. HE treatment began from 9th week until week 17th. **b** Body weight, and **c** Weight gain (15 weeks) and **d** trajectories of the adipose tissue weight of each treatment group are shown [all in gr]. **e** Representative images of H&E staining of the visceral adipose tissue section (at least 5 parts were evaluated for each sample) and **f** white adipose tissue (WAT) adipocyte size measurement (performed in triplicate). HE: herbal extract; ND: normal diet; HFD: high AGEs-fat diet; HD: high dose; LD: low dose; MD: medium dose; DSS: 2.5% dextran sulphate sodium. Data as mean with 95% CI, n = 6 per group. ** represents *p* < 0.01 (significant difference between the groups)

Four-week C57BL/6 male mice (Royan Institute for Biotechnology, Esfahan, Iran) acclimatized in animal facility under standard conditions (temperature 23–24 °C, humidity 32–33%, light/dark cycle of 12 h/12 h) and fed with pellet diets and water ad libitum (intake measured daily). The study protocol was approved by the Ethics Committee of the Royan Institute (IR.SSU.MEDICINE.REC.1396.164). All experiments were performed in accordance with relevant guidelines and regulations. Sample size was calculated as ≥ 5 mice/group assuming α = 0.05, ß = 0.2 and standard deviation (SD = 1.14, as the highest observed dispersion). In the first step, mice were randomized into two dietary groups (n = 48/group) fed either ND or HFD (see Additional file 1: Table S1). In the second step, mice receiving both ND and HFD were divided into 8 groups (n = 6/group) groups following an identical treatment format (Fig. 1a) (see Additional file 1: Table S2). Henceforth, we refer to the High AGEs-Fat Diet as High-Fat Diet (HFD).

The body weight of each mouse was measured weekly. One day before sacrifice, feces samples were collected. On the last day of 17th week, animals were fasted for 6 h and sacrificed. Blood (for plasma) was collected by cardiac puncture and the spleen, adipose tissue and colon were dissected and snap-frozen in liquid nitrogen. Approximately 1 cm of the distal colon was sampled for histological examination. The remaining colonic tissue, containing both inflamed and non-inflamed areas, was rinsed in PBS and frozen in liquid nitrogen. All collected materials were stored in − 80C until analysis.

### Assessment of colitis and disease activity index (DAI)

The body weight loss was compared to the initial body weight (scored as 0–4). Fecal consistency (scored as 0–4) and blood in the stool (scored as 0–4) were assessed throughout the DSS treatment and recovery period. The final DAI score combines all three criteria [30].

### Myeloperoxidase (MPO) assay

Colon MPO activity, a marker of neutrophilic infiltration, was assessed using MPO assay kit (NampoxTM, Navand Salamat Co., Iran), according to the protocol. A proximal colon sample from each mouse was excised, immediately rinsed with ice-cold saline, blotted dry and frozen at − 80 °C. Subsequently, the tissue samples were thawed, weighed and homogenized in sample buffer 1 × (pH: 6.0) containing 0.5% hexadecyl trimethyl ammonium bromide. Samples were centrifuged at 10,000 rpm for 10 min in 4 °C. A microplate reader (Lab system Multiskan EX, Helsinki, Finland) was used to measure the changes in absorbance at 450 nm in supernatant using 3,3′,5,5′-tetramethylbenzidine (TMB) and H2O2. The results were reported as the U/mg tissue [31].

### Histologic examination [Hematoxylin–eosin (H&E) staining and immunohistochemistry]

Distal part of colon tissue samples were rinsed with PBS, fixed in 10% formalin for 24 h and embedded in paraffin, sliced to 5 μm-thickness sections, and stained with H&E following routine protocol. Finally, slides were observed under a microscope. For each sample, six horizons were selected to assess the colonic inflammation, which was denoted as histological scores (HS), according to the reported criterion [32].

Five-micrometer thick sections were cut from the colon tissue blocks, dewaxed and rehydrated. Tissue specimen were mounted on Vectabond coated (Vector Labs, Burlingame, California, USA) glass slides. The slides were frozen and stored at − 20 °C until further processing. Macrophages were identified by staining tissue sections with commercially available antibody to macrophage marker, CD68 (concentration 1:200). Prior to primary antibody staining, slides were treated with 10% swine serum for 20 min. Primary antibody or isotype control were incubated for 1 h. Slides were treated with biotinylated secondary antibody for 20 min and NovaRED (Vector Labs, UK) reagent to visualize antibody staining for 5 min. Slides were then counterstained with hematoxylin (Dako, USA), mounted, and cover slipped. Three random photomicrographs of each slide at 40 × magnification were taken under Nikon Eclipse E600 microscope (Japan). The photographer was blinded to the stain and patient diagnosis. Photomicrographs were presented to a panel of 3 blinded graders with quantified positive staining and determined the mean number of positive cells per high-powered field [33].

### Determination of cholesterol, triglycerides, HDLs, LDLs and blood glucose levels

For biochemical analysis, plasma was obtained from blood by centrifugation at 8000 rpm for 10 min at 4 °C. EDTA was used as an anticoagulant. Plasma total cholesterol (T-CHO), high density lipoproteins (HDLs), low density lipoproteins (LDLs) and triglycerides (TGs) were measured using commercial diagnostic kits (Pars Azmoon kit, Pars Azmoon Inc., Iran), and the plasma glucose level was determined by glucometer after 6 h fasting.

### Terminal deoxynucleotidyl transferase–mediated dUTP nick end labeling assay

Terminal deoxynucleotidyl transferase–mediated dUTP end labeling (TUNEL) assay was done to picture apoptotic cells. Five μm thick sections were cut from the colon tissue blocks, deparaffinized and rehydrated. Then slides were fixed with 4% methanol-free paraformaldehyde in phosphate-buffered saline (PBS) for 10 min at room temperature. After fixation, slides were washed with PBS and underwent permeabilization with 20 µg/mL of proteinase K solution for 5 min, then washed twice in PBS. Then 100 μL of equilibration buffer was added at room temperature for 5–10 min. Samples were washed with PBS and then incubated with terminal deoxynucleotidyl transferase, recombinant (rTdT) buffer at 37 °C for 60 min inside the humidified chamber according to the manufacturer’s protocol (Promega, USA). Reaction was terminated by adding 2 × SSC for 15 min. The slides were washed thrice, using PBS, for 5 min to remove unincorporated fluorescein-12-dUTP nucleotides. Detection of fragmented DNA was examined under a fluorescence inverted microscope (Carl Zeiss, Germany). For each sample, the total number of cells and the number of TUNEL-positive cells were quantified in 10 representative fields. The results are presented as a representation from a series of three separate experiments [34].

### qRT-PCR analysis

Total RNA was isolated using TRIzol reagent (Ambion, USA) and purified by LiCl (Sigma, USA), then underwent DNase treatment using the RNase-free DNase Set (TaKaRa, Japan). Then RNA was reverse transcribed using M-MLV Reverse Transcriptase (TaKaRa) according to manufacturer’s instructions, and resulting cDNA was amplified by qPCR using predesigned primers, and SYBR Green PCR Master Mix (Japan) as described elsewhere. Oligonucleotide primer sequences are shown in Additional file 1: Table S3.

### Immunoblotting analysis

Tissues were lysed using TRI reagent (Thermo Scientific, USA), according to the manufacturer protocol. Equal amounts of each protein sample (30 μg) were separated by SDS-PAGE and transferred to PVDF membranes (Bio Rad, USA). After blocking the membranes with 10% skim milk or bovine serum albumin (BSA) (Millipore, USA), membranes were incubated with different primary antibodies for 2 h at room temperature. Primary antibodies were rabbit anti-RAGE antibody (1:2000, Abcam, AB3611, UK), anti-P65 antibody (1:500, Cell Signaling, 4764S, USA), anti-sirt1 (1:1000, Abcam, AB110304), anti-PPARγ (1:5000, Santa Cruz, SC7273, USA) antibody and mouse anti GAPDH antibody (1:5000, Santa Cruz). Then, membranes were incubated for 1 h at room temperature with an appropriate secondary antibody: horseradish peroxidase (HRP)-conjugated goat anti mouse IgG (1:5000, Dako, P0447, Denmark), or HRP-conjugated goat anti-rabbit IgG (1:16,000, Santa Cruz, SC2301). HRP-conjugated IgG bound to each protein band was visualized by an Amersham ECL Advance Western Blotting Detection Kit (GE Healthcare, USA). The intensity of each band was quantified by Image J software.

### Statistical analysis

Kolmogorov–Smirnov test was used for assessing the data distribution in all datasets. Results are presented as mean and 95% confidence interval (CI) in bars overlaid with scatter plot for maximal transparency. Data were obtained on triplicate data sets for each sample and analyzed by Kruskal–Wallis and Mann–Whitney test for post-hoc (non-parametric) comparisons. All statistical analyses were performed using SPSS (version 17.0) and GraphPad Prism Software (Version 8.0a Graph Pad Software Inc., USA). *P*-value < 0.05 was considered to be statistically significant. Power analysis was done by PASS-NCSS (version 11).

## Results

### Chemical composition and stability of HE

There was no difference between curcumin and other component before and after the end of study attesting to the stability of the HE during the study period. The phytocomponents of HE were recognized by GC–MS analysis. Additional file 1: Table S4 provides a list of the active compounds, their retention time (RT), quantity, molecular formula (MF), nature, and biological activity. Also, curcumin amount was measured by UV-spectrophotometry: 140–160 mg (27.61%) per 500 mg of HE. The two most abundant HE components were (1) terpenes (24.36%), and (2) curcumin (27.61%). Several other compounds with proven anti-inflammatory effects were in the range of 5–11%: NSAIDs (Nonsteroidal anti-inflammatory drugs) (6.368%), cyclic ethers (7.91%), artemiseole (8.854%), and limonene dioxide (11.247%).

### HE reduced HFD induced changes in the body/fat weight gain and in the modified lipid profile

The body weight in HFD and ND-fed mice was monitored for 15 weeks. HE consumption decreased the body weight in HFD mice; by approximately 49% in the higher dose and 7% in ND group (Fig. 1b, c). Moreover, HFD-fed mice had higher white adipose tissue (WAT) weight than ND mice. HE caused a 40% reduction in WAT weight (Fig. 1d). HFD also increased concentration of circulating glucose, TG, CHO, HDL and LDL (*p* < 0.05). HE strongly reversed this effect (Table 1) and simultaneously induced a decrease of the visceral adipocyte sizes (by 47% in the high-dose mice; Fig. 1e, f). Henceforth, we refer to the HE (wherever the dose of the HE is not mentioned) as high dose (HD) of HE.

**Table 1:** Effect of oral herbal extracts on Blood parameters

Blood Biochemical indexes (mg/dl)	Groups			% Difference between HFD and HFD HE-HD (%)
	HFD	ND	HFD HE-HD	
T-CHO	281.81 ± 3.2^*^	34.82 ± 1.2	160.71 ± 6.2	43
TGs	287.7 ± 4.2^*^	22.2 ± 1.1	150.23 ± 3.2	48
HDLs	3.98 ± 0.5^*^	1.92 ± 0.3	2.26 ± 0.4	43
LDLs	6.44 ± 0.45^*^	3.27 ± 0.2	4.64 ± 0.3	28
FBS	270^*^	98	100	63

HE: herbal extract; ND: normal diet; HFD: high AGEs-fat diet; HD: high dose; FBS: fast blood glucose; HDLs: high density lipoproteins; LDLs: low density lipoproteins; ND: normal diet; T-CHO: Plasma total cholesterol; TGs: triglycerides. * represents significant difference with ND parameters at *p* < 0.05 (significant difference between HFD and HFD HE-HD group)

### HE reduced DSS-induced colitis, inflammation, oxidative stress and MPO activity

**Fig. 2:**
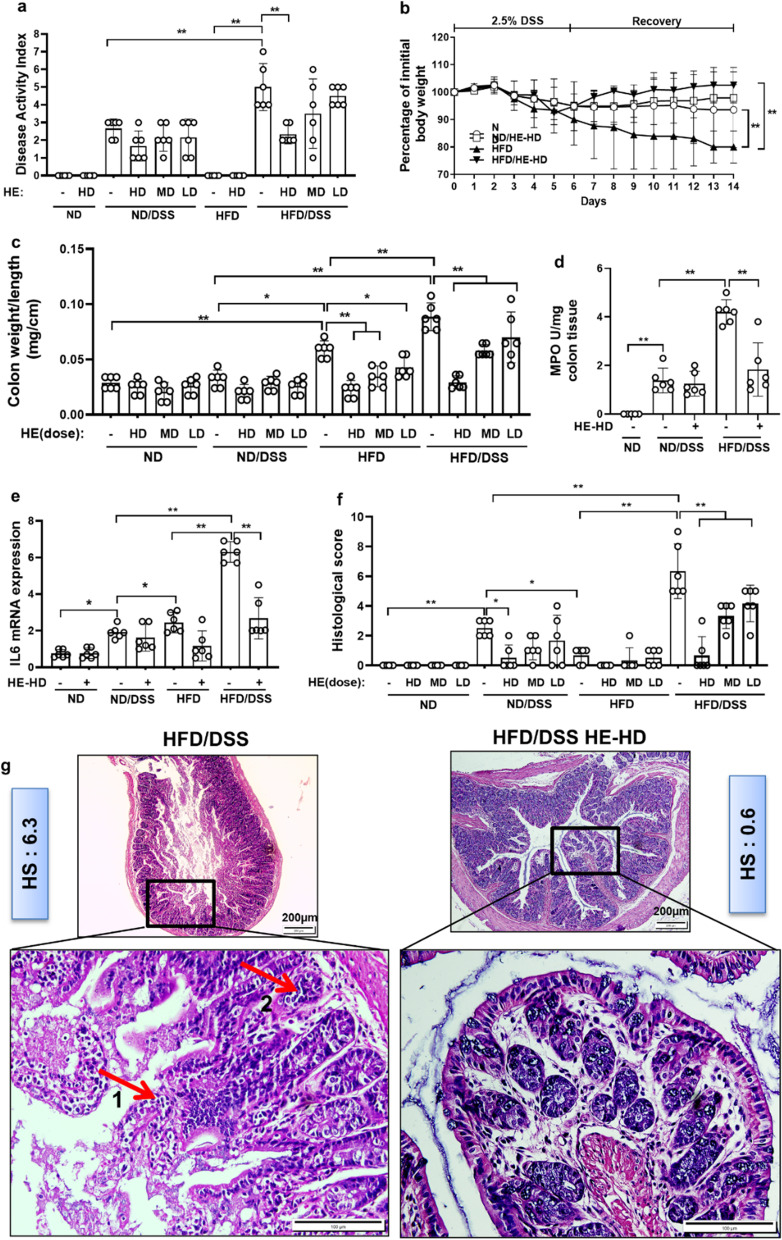
DSS-induced colitis, inflammatory mediators and histological score. **a** Disease activity index, **b** Percentage of initial body weight, **c** colon weight to length, **d** MPO activity, **e** mRNA expression of *IL-6*, **f** Histological score (at least 5 parts were evaluated for each sample), and **g** Representative images of H&E staining of distal colonic sections: red arrow 1 shows neutrophils and red arrow 2 indicate cryptitis. HE: herbal extract; ND: normal diet; HFD: high AGEs-fat diet; HD: high dose; LD: low dose; MD: mid dose; DSS: 2.5% dextran sulphate sodium. HS: histological score; MPO: myeloperoxidase. Data as mean with 95% CI, n = 6 per group. * and ** respectively represents *p* < 0.05 and *p* < 0.01 (significant difference between the groups)

Since obesity is a strong risk factor for colitis, we implemented DSS to induce colitis in obese HFD mice and test whether HE can ameliorate it. 7-day long administration of 2.5% DSS to HFD-fed mice resulted in a distinct colitis phenotype at week 16th confirmed by an increase of DAI, *IL-6* mRNA expression and MPO activity (see Additional file 1: Fig. S1). The HFD mice showed a similar weight loss during DSS treatment regardless of HE doses. In contrast, HFD mice with colitis administered HE were in an overall superior state in the recovery phase (week 17; vs. ND mice) and their DAI score was reduced by approximately 3 points (Fig. 2a, b). HE reduced colon weight to length ratio in HFD-fed mice by 70% (Fig. 2c). MPO activity in the colon increased 3.2-fold after DSS administration in HFD mice but was ameliorated by 2.3-fold by HE treatment (Fig. 2d). Furthermore, *IL-6* expression in HFD/DSS was 3.6-fold higher than in ND/DSS group. HE reduced *IL-6* gene expression in the colon of HFD/DSS mice by 2.3-fold (Fig. 2e).

### HE reduced HFD-dependent colonic histopathophysiology in DSS-induced colitis

H/E and CD68 staining demonstrated an increased infiltration of neutrophils and macrophages in the colon of HFD/DSS mice; HE reduced histological score by 2.3-fold (Fig. 2f, g) (see Additional file 1: Fig. S2). In parallel, histological changes of the colonic mucosa showed an acute and chronic colitis in HFD/DSS mice as indicated by high neutrophil and histiocytes population, damaged villi, decreased number of goblet cells and crypts, cryptitis, hyper-chromatic nuclei of the gland cells and loss of the crypt architecture. These changes were less pronounced in ND/DSS mice and reduced in HE treatment group by 10.5-fold (see Additional file 1: Fig. S3). Compared with either low or medium-dose, high-dose HE was associated with more attenuation in inflammation, oxidative stress and histological changes (Fig. 2f, g).

### HE reduced circulating IL6 and prevented enlargement of the spleen

**Fig. 3:**
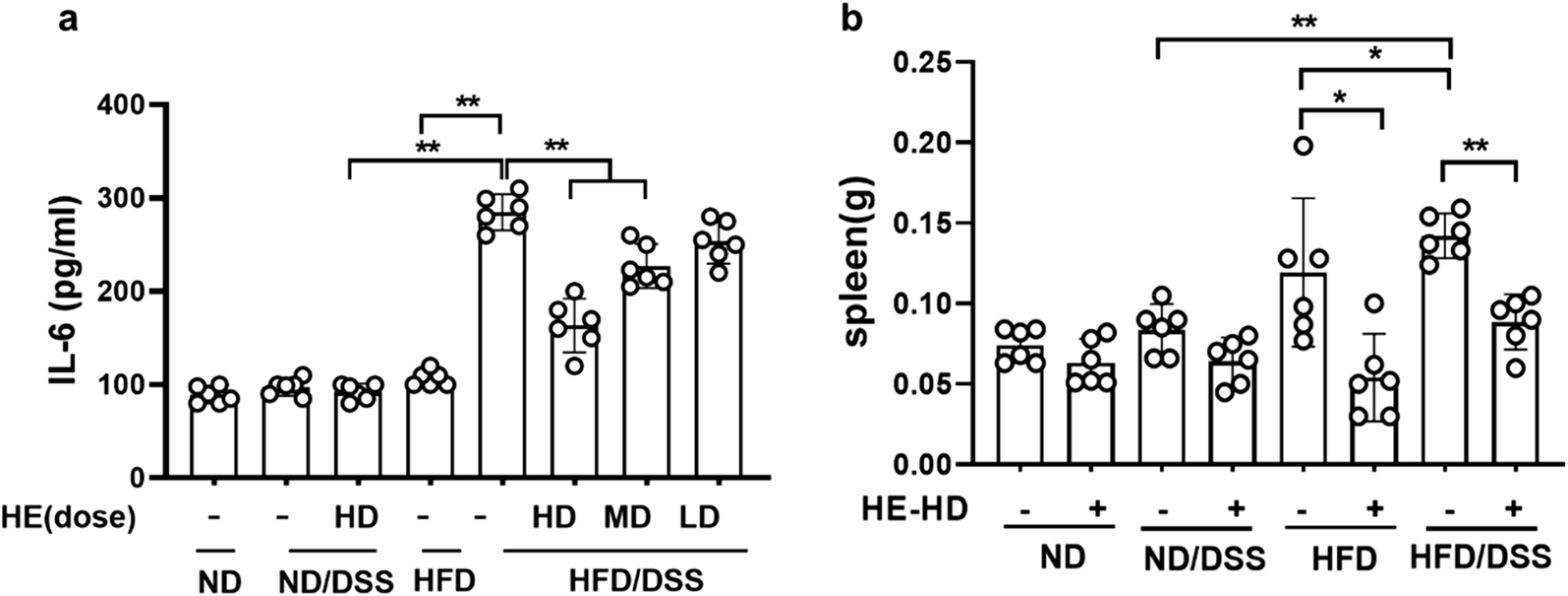
Circulating IL-6 and the spleen weight. **a** pro-inflammatory cytokine IL-6 in plasma, **b** spleen weight. HE: herbal extract; ND: normal diet; HFD: high AGEs-fat diet; HD: high dose; LD: low dose; MD: medium dose; DSS: 2.5% dextran sulphate sodium. Data as mean with 95% CI, n = 6. * and ** respectively represents *p* < 0.05 and *p* < 0.01 (significant difference between the groups)

DSS treatment of HFD-fed mice increased circulating IL-6 by threefold (vs ND mice) and HE treatment ameliorated it by 1.7-fold. Additionally, in DSS-treated mice, the spleen weight increased by 1.7-fold in HFD-fed mice (versus ND mice). HE treatment effectively prevented the spleen weight increase in DSS-induced colitis mice (Fig. 3a, b).

### HE reduced RAGE, NF-κB expression and modulated Sirt1 and DDOST (AGER1) expression in the colon

**Fig. 4:**
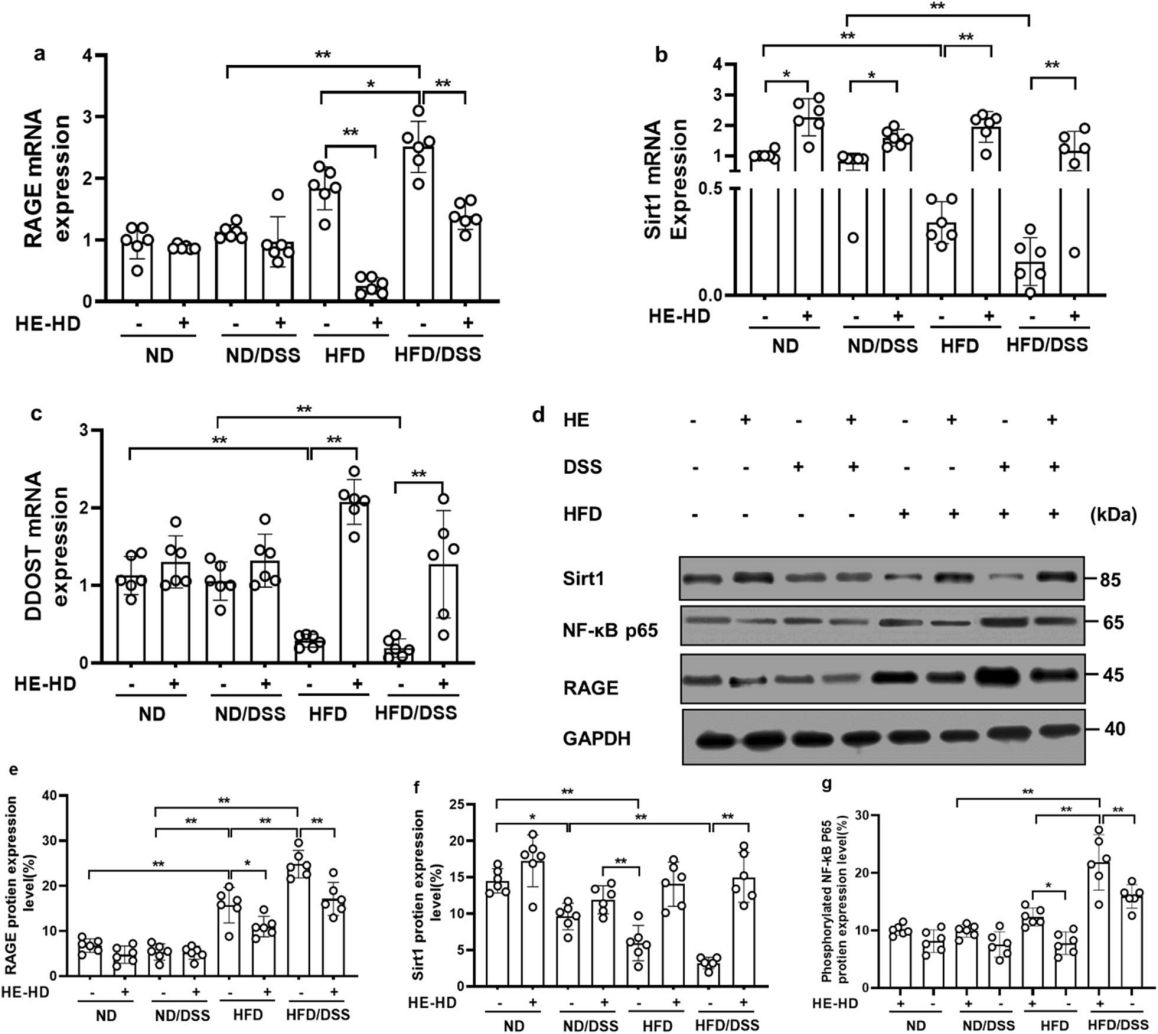
RAGE, Sirt1, NF-κB p65 and DDOST (AGER1) changes in the colon. **a** *RAGE*, **b** *Sirt1*, **c** *AGER1* (*DDOST*), **d** Representative immunoblotting bands (performed in triplicate) and **e**–**g** quantification of RAGE, Sirt1 and NF-κB p65 protiens. HE: herbal extract; ND: normal diet; HFD: high AGEs-fat diet; HD: high dose; DSS: 2.5% dextran sulphate sodium; AGEs: Advanced glycation end products; *AGER1*: Advanced glycation end products receptor 1; *DDOST*: dolichyl-diphosphooligosaccharide-protein glycosyltransferase subunit; NF-κB: nuclear transcription factor kappa B; *RAGE*: advanced glycation end products receptor. Data as mean with 95% CI, n = 6 per group. * and ** respectively represents *p* < 0.05 and *p* < 0.01 (significant difference between the groups)

We detected an up-regulation of *RAGE* in HFD/DSS mice (compared to ND/DSS mice) at both RNA (by 1.6-fold; Fig. 4a) and protein (by 2.4-fold; Fig. 4d, e). In HFD/DSS mice (versus HFD/DSS/HE mice), HE reduced *RAGE* activation at both RNA (by 1.8-fold) and protein (by 1.4-fold); this was paralleled by a decrease in the downstream factor NF-κB by 1.3-fold in HFD/DSS/HE mice. Furthermore, HE increased mRNA expression of *AGER1* in the HFD-fed DSS-colitis mice by 6.6-fold (Fig. 4c).

In order to establish the long-term effects of HFD on inflammation, the *RAGE* and *IL-6* expressions were assessed in HFD mice after 32 weeks; both *RAGE* and *IL-6* were down-regulated (Additional file 1: Fig. S4).

*Sirt1* is down-regulated by 5.7-fold in HFD/DSS mice (compared to ND/DSS mice). HE treatment increase the expression of *Sirt1* by approximately 7.7-fold at RNA and 4.6-fold at protein level (Fig. 4b, d, f). This consequently decreased activation of Sirt1 downstream target protein, p65, by fourfold in the HFD/DSS mice (Fig. 4d, g).

### HE reduced apoptosis in the colon

**Fig. 5:**
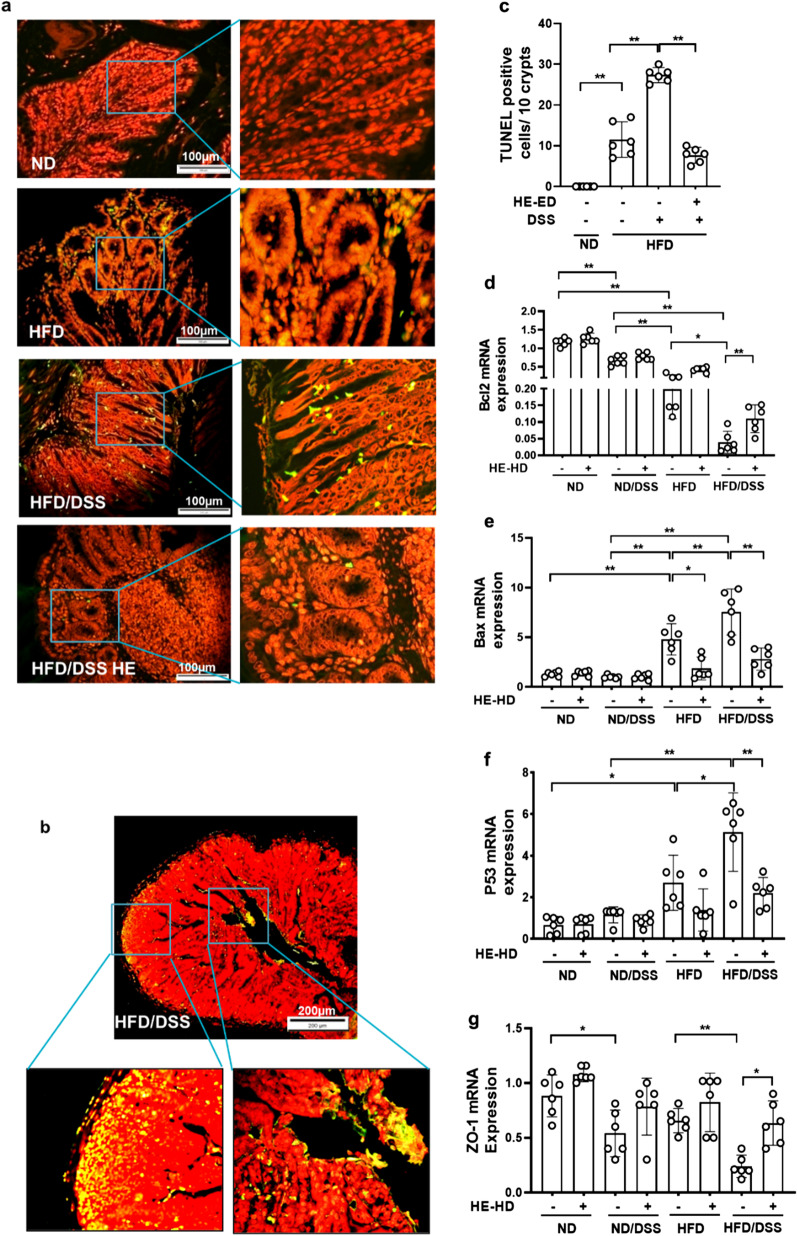
Apoptosis in the colon. **a** and **b** Apoptotic epithelial cells in the colon crypts are shown, **c** the numbers of TUNEL-positive cells in the colon (at least 5 parts were evaluated for each sample). RNA levels of **d** *Bcl2*, **e** *Bax*, **f** *P53*, **g** *ZO-1*. HE: herbal extract; ND: normal diet; HFD: high AGEs-fat diet; HD: high dose; DSS: 2.5% dextran sulphate sodium; *Bax*: BCL2 Associated X, Apoptosis Regulator; *Bcl2*: BCL2 Apoptosis Regulator; *P53*: Tumor Protein P53; TUNEL: terminal deoxynucleotidyl transferase–mediated dUTP end labeling; *ZO-1*: zonula occludense-1. Data as mean with 95% CI, n = 6 per group. * and ** respectively represents *p* < 0.05 and *p* < 0.01 (significant difference between the groups)

HE may prevent apoptosis and maintaining tight junctions and protecting barrier function in the gut. TUNEL staining demonstrated that the apoptosis in the colonic epithelium (crypts) and smooth muscle cells increased by 2.4-fold in HFD/DSS group compared to HFD mice. HE treatment ameliorated apoptosis by 3.5-fold (Fig. 5a–c). As shown in Fig. 5d, down-regulation of (anti-apoptotic) *Bcl-2* and up-regulation of (pro-apoptotic) *Bax* factor were observed in HFD and HFD/DSS groups. By contrast, HE markedly augmented the expression of *Bcl-2* (by 2.9-fold) and attenuated the expression of *Bax* in the colon by 2.7-fold (Fig. 5e) in HFD/DSS mice. RNA level of *ZO-1* was down-regulated in ND/DSS (by 1.7-fold) and HFD/DSS (by 2.8-fold) group compared to ND and HFD groups. Interestingly, the *ZO-1* gene was up-regulated by HE in HFD/DSS mice by 2.6-fold (Fig. 5g).

P53 is a key molecule involved in apoptosis and patients with UC have an elevated p53 mutation load in noncancerous colon epithelial cells [35]. A fourfold increase in *p53* transcript was found in HFD group compared to ND-fed mice. In HFD/DSS mice (compared to ND/DSS) *p53* elevation was fourfold higher. HE treatment reversed this increase by 2.3-fold demonstrating its suppressive effect on apoptosis and inflammation (Fig. 5f).

## Discussion

Thermal processing and food storage are the main generators of AGEs products. HFD diet increases risk of obesity, diabetes, hypertension, cardiovascular disease and other diseases. Consumption of HFD increases the exposure to food AGEs, triggering a persistent low-grade inflammation in the colon and changes in gut microbiota. This in turn increases the intestinal permeability to the microbial end-products and AGEs that are associated with intestinal inflammation and promotion of cancer [36–40].

Murine DSS-induced colitis is similar to the human UC phenotype [41]; the common mechanistic features include neutrophil and monocyte infiltration and their activation by inflammatory mediators and cell adhesion molecules [42]. Neutrophils produce pro-inflammatory cytokines enhancing oxidative stress that provoke colitis [32, 43]. Westernized diet intensifies immune cell infiltration as indicated by elevated MPO activity [44] and we show that DSS induction enhanced neutrophil and monocyte infiltration and MPO activity in the colon of HFD mice. The design and the two-hit model used in our study were constructed to reflect the clinical scenario with maximal fidelity. We first utilized HFD to increase colon susceptibility (first hit) and generated pronounced colitis by adding DSS (second hit). We also used a long-term HFD exposure to ensure that pathophysiological alterations developed in a chronic rather than rapid, acute process. This was matched by a similarly protracted exposure to treatment to allow a progressive restoration of the gut homeostasis—an approach typical for treatment of chronic gastro-intestinal tract derangements and for the use of herbal products in particular. The male sex was chosen given that female mice are partially protected against chemically induced colitis [45].

*RAGE* and *DDOST* are expressed in a variety of cells including endothelial, epithelial, neural, smooth muscle, immune-inflammatory cells and enterocytes. *RAGE* triggers pro-inflammatory pathways such as NF-κB, But *DDOST* facilitates degradation of AGEs and scavenging it from plasma, resulting in reduction of ROS generation and RAGE down-regulation. NF-κB itself modulates expression of *RAGE* that maintains and boosts the signal [7, 18]. In CD patients, up-regulation of *RAGE* was observed [18–20]. Although *RAGE* increased both in HFD and DSS-treated groups, its elevation was maximal in HFD/DSS mice. HE effectively decreased *RAGE*; an effect concurrent with attenuation of the colitis symptoms. The most abundant component of HE (ginger, turmeric, boswellia and cat’s claw) were terpenes and curcumin that may be the most important elements that prevent colitis in this study. Recent studies showed that turmeric, ginger and boswellia consumption modified gut microbiota leading to an improvement of intestinal epithelium integrity by reducing metabolic endotoxemia in DSS-induced colitis [10, 46, 47]. In line with our data, Malapel et al. demonstrated *RAGE* up-regulation during inflammation in the colon; over-activated *RAGE* induced intestinal inflammation by promoting oxidative stress and endothelial activation that was halted by FPSZM (a specific *RAGE* inhibitor). Simultaneous expression of *RAGE* in immune and non-immune cells is important for colitis development consistent with a massive infiltration during intestinal inflammation [18]. Moreover, Pongrats et al. revealed that HFD has an immunosuppressive effect in ageing Wistar rats, by decreasing *IgM*, and *IL-1β* when compared to normal diet (ND) [48]. This is also in accordance with our results; 32-week long consumption of HFD decreased colonic *RAGE* and *IL-6* expression. Colitis is also associated with reduction of *Sirt1* expression and elevation of NF-κB activation [49]; curcumin was shown to reverse them [12] by promoting Sirt1 signaling activation [50]. AGE-RAGE interaction stimulates ROS formation and *Sirt1* reduction leading to an increased inflammation [26]. We demonstrate that HFD increased activation of NF-κB in the colon and secretion of IL-6 in both plasma and colon, both of which were down-regulated by HE. Additional research to clarify the association between *AGER1*, *RAGE*, *Sirt1*, HFD and colitis is warranted.

Our study reveals for the first time that a long-term HFD decreased *AGER1* expression in the colon and HE prevented this reduction (in HFD mice). It is hypothesized that *AGER1* is expressed more likely in the resident and/or infiltrating immune cells rather than non-immune cells of the colon. In healthy individuals, elevated levels of AGEs and oxidative stress in the circulation increase the expression of *AGER1* (*DDOST*) and consequently reduction of RAGE expression and oxidative stress. Our data showed that 2 months did not elevate *RAGE* expression, while 4 months of HFD reduced *AGER1* and increased *RAGE*; this is consistent with previous studies which showed that HFD did not provoke inflammation and oxidative stress in *AGER1* transgenic mice. It appears that prolonged consumption of HFD in obesity and diabetes down-regulates the *AGER1* expression. In contrast, restricted amount of AGEs diet in mice maintained *AGER1* in a normal range and inhibited elevated (aging-related) oxidative stress [51]. Suppression of *AGER1* in peripheral blood mononuclear cells (PBMCs) was partially restored after receiving low-AGEs diet intervention in individuals with T2D [7]. Moreover, *DDOST* is used in diagnostic microarray for IBD given that it is overexpressed in PBMCs of UC patients [24]. While there is an reverse association between *AGER* versus *RAGE* and their results on oxidative stress in both human and mice, a precise role for *AGER1* (as a scavenging receptor) in diminishing the deleterious effect of AGEs is unclear [7]. Previous reports demonstrated that curcumin, boswellia and ginger have AGEs-trapping and anti-obesity capacity. However, little is known regarding the *AGER1* role in etiology of colitis. Interestingly, Graham and colleagues [22], demonstrated that *TMEM258* is a required component of oligosaccharyl transferase complex essential for N-linked protein glycosylation. Homozygous deficiency of Tmem258 in colonic organoids resulted in an unresolved endoplasmic reticulum (ER) stress culminating in apoptosis. *TMEM258* is a central mediator of ER quality-control and intestinal homeostasis.

Activation of the RAGE pathway regulates apoptosis and may lead to tissue damage in UC and CD [19, 35]. The intestinal epithelial layer acts as a pathogen barrier; an impaired apoptosis decreases the epithelial integrity and contributes to mucosal inflammation and carcinoma. It has been found that patients with active UC who need surgery have higher apoptotic indices and CD68 staining than UC patients who were receiving medication [52]. The epithelial barrier can disintegrate by epithelial cell apoptosis and/or tight junction loss [27]. A rat study demonstrated a decrease of Occludin and *ZO-1* expression and increased intestinal permeability under high-AGE diet [53]. We found that down-regulation of *Bcl2*, and *Bax* and *p53* up-regulation altogether increased apoptosis in HFD mice; the effect was most pronounced in HFD/DSS group. This implies that exposure to AGEs and (and subsequently obesity) increases susceptibility to colitis by increasing apoptosis in the intestinal epithelial cells.

## Conclusion

Summarizing, our results demonstrate that protracted administration of ginger, turmeric, boswellia and cat’s claw effectively alleviated inflammation triggered by the HFD-induced obesity and colitis. This study for the first time demonstrated that long-term HFD decreased *AGER1* level and had immunosuppressive effect in the colon. The beneficial effect of herbal extract can be attributed to the modulation of pro-inflammatory cytokine gene expression (i.e. *RAGE*, *AGER1*, *Sirt1* and NF-κB) and subsequent attenuation of the clinical symptoms.

## Supplementary Information

**Additional file 1**. **Table S1**. Nutrients content of diets (HFD and ND). **Table S2.** Study groups. **Table S3.** Primer sequences used in this study. **Table S4.** Phytocomponents identified in the HE by GC-MS. **Figure S1.** Determination of optimal concentrations of DSS dose and diet duration. **Figure S2.** Immunohistochemical staining of macrophages in colon. **Figure S3.** Representative images of H&E staining of distal colonic sections. **Figure S4**. Comparison of 4 and 8 months HFD consumption.

## Data Availability

The data and materials that support the findings of this study are available from the corresponding author upon reasonable request.

## Abbreviations

AGEs: Advanced glycation end products
AGER1: Advanced glycation end product receptor 1
ANOVA: One-way analysis of variance
BSA: Bovine serum albumin
CD: Crohn’s disease
DSS: Dextran sodium sulfate
DAI: Disease activity index
DDOST: Dolichyl-diphosphooligosaccharide-protein glycosyltransferase subunit
FBS: Fast blood glucose
HE: Herbal extracts
HFD: High AGEs-fat diet 60%
HD: High dose
HDLs: High density lipoproteins
H&E: Hematoxylin-eosin
HRP: Horseradish peroxidase
IBD: Inflammatory bowel disease
LDLs: Low density lipoproteins
LD: Low dose
MD: Mid dose
MF: Molecular formula
MPO: Myeloperoxidase
ND: Normal diet
NF-κB: Nuclear transcription factor kappa B
NIST: National Institute of Standards and Technology
NSAIDs: Nonsteroidal anti-inflammatory drugs
OST48: Oligosaccharyltransferase subunit 48
PBS: Phosphate-buffered saline
RT: Retention time
rTdT: Terminal deoxynucleotidyl transferase, recombinant
SCFA: Short chain fatty acids
SEM: Standard error of mean
Sirt1: Silent mating type information regulation-1
T-CHO: Total cholesterol
TGs: Triglycerides
TMB: 3,3′,5,5′-Tetramethylbenzidine
TUNEL: Terminal deoxynucleotidyl transferase–mediated dUTP end labeling
UC: Ulcerative colitis
UV: Ultraviolet
VSC: Visceral
WAT: White adipose tissue
