# Supplementary material for: Anti-inflammatory and -apoptotic effects of a long-term herbal extract treatment on DSS-induced colitis in mice fed with high AGEs-fat diet
Source: Nutr Metab (Lond). 2021 Aug 11;18:77. doi: 10.1186/s12986-021-00603-x (PMC8359107; doi:10.1186/s12986-021-00603-x)
Supplement: Supplementary file 1 — Additional file 1. Table S1. Nutrients content of diets (HFD and ND). Table S2. Study groups. Table S3. Primer sequences used in this study. Table S4. Phytocomponents identified in the HE by GC-MS. Figure S1. Determination of optimal concentrations of DSS dose and diet duration. Figure S2. Immunohistochemical staining of macrophages in colon. Figure S3. Representative images of H&E staining of distal colonic sections. Figure S4. Comparison of 4 and 8 months HFD consumption. [file 12986_2021_603_MOESM1_ESM.doc]

**Running title: An herbal extract diminishes DSS-induced colitis**

**Title: Anti-inflammatory and -apoptotic effects of a long-term herbal extract treatment on DSS-induced colitis in mice fed with high AGEs-fat diet**

Fatemeh Azizian-Farsani^1^, Marcin Osuchowski^2,≠^, Navid Abedpoor^3^, Farzad Seyed Forootan^3,4^, Maryam Derakhshan^5^, Mohammad Hossein Nasr-Esfahani^3,#^, Mohammad Hasan Sheikhha^1,6,±^, Kamran Ghaedi^7,#^

^1^ Department of Medical Genetics, Shahid Sadoughi University of Medical Sciences, Yazd, Iran., ^2^ Ludwig Boltzmann Institute for Clinical and Experimental Traumatology in AUVA Research Center, Vienna, Austria., ^3^ Department of Cellular Biotechnology, Cell Science Research Center, Royan Institute for Biotechnology, ACECR, Isfahan, Iran., ^4^ Legal Medicine research Center, Legal Medicine Organization, Tehran, Iran., ^5^Department of Pathology, Isfahan University of Medical Sciences, Isfahan, Iran., ^6^ Biotechnology Research center, International Campus, Shahid Sadoughi University of Medical Sciences, Yazd, Iran., ^7^Department of Cell and Molecular Biology and Microbiology, Faculty of Biological Science & Technology, University of Isfahan, Isfahan, Iran.,

**Corresponding authors:**

^#^ Professor. Kamran Ghaedi. Department of Cell and Molecular Biology and Microbiology, Faculty of Biological Science and Technology, University of Isfahan, Hezar Jerib Ave., Azadi Sq., P.O. Code 81746-73441, Isfahan, Iran. Phone no: +98-31-37932479; Fax no: +98-31-37932456. Emails: kamranghaedi@sci.ui.ac.ir, [kamranghaedi@yahoo.com](mailto:kamranghaedi@yahoo.com), ± Dr. Mohammad Hasan Sheikhha. Department of Medical Genetics, Shahid Sadoughi University of Medical Sciences, Yazd, Iran. Phone no: +98 913 357 7387; [sheikhha@yahoo.com](mailto:sheikhha@yahoo.com) (Mohammad Hasan Sheikhha), ^≠^ Dr. Marcin Osuchowski. Ludwig Boltzmann Institute for Clinical and Experimental Traumatology in AUVA Research Center, Vienna, Austria. Phone no: +43-1-33110469, Fax no: +43-1-33110 460. [marcin.osuchowski@trauma.lbg.ac.at](mailto:marcin.osuchowski@trauma.lbg.ac.at), # Professor Mohammad Hossein Nasr-Esfahani, Department of Cellular Biotechnology, Cell Science Research Center, Royan Institute for Biotechnology, Royan, Salman Streets, P.O. Code 816513-1378, Isfahan, Iran. Email: mh.nasr-esfahani@royaninstitute.org. Tel: +98 31 95015694, Fax: +98 31 95015688.

**Supplementary table**s:

| **Table S1. Nutrient content of diets (HFD and ND)** | | | |
| --- | --- | --- | --- |
|  | **HFD** | **Normal** |  |
| Protein (g %) | 25.6 | 26 |  |
| Carbohydrate (g %) | 26.6 | 50.3 |  |
| Fat (g %) | 35 | 5. 35 |  |
| Calories (kcal/g) | 5.2 | 3.4 |  |
| Total AGEs (units/mg) | 995.4 | 117.4 |  |
| Fat-associated AGEs (units/mg) | 329.6 | 1.4 |  |
| Maltodextrin (g %) | 125 | 25 |  |
| AGEs: Advanced Glycation End Products, HFD: High AGEs-Fat diet, and ND: Normal diet | | |  |

| **Table S2. Study Groups.** | | | | | | | | |
| --- | --- | --- | --- | --- | --- | --- | --- | --- |
| **Diet** | **ND** | | | | **HFD diet** | | | |
| **DSS** | **-** | | **+** | | **-** | | **+** | |
| **Herbal Extract** | **-** | **+** | **-** | **+** | **-** | **+** | **-** | **+** |
| **Groups with Different**  **doses** | ND | ND/ HE-LD | ND/DSS | ND/DSS/HE-LD | HFD | HFD/ HE-LD | HFD/DSS | HFD/DSS/ HE-LD |
|  |  | ND/ HE-MD |  | ND/DSS/HE-MD |  | HFD/ HE-MD |  | HFD/DSS/ HE-MD |
|  |  | ND/ HE-HD |  | ND/DSS/HE-HD |  | HFD/ HE-HD |  | HFD/DSS/ HE-HD |
| HE: herbal extract; ND: normal diet; HFD: high AGEs-fat diet; HD: high dose (0.6 mg/g); LD: low dose (0.15 mg/g); MD: mid dose (0.3 mg/g); DSS: 2.5% dextran sulphate sodium (MW around 40,000 g/mol); – : (getting HE); + : ( not getting HE). | | | | | | | | |

| **Table S3. Primer sequences used in this study** | |
| --- | --- |
| **Gene** | **Sequence (5`-3`)** |
| *GAPDH* | forward: TGCCGCCTGGAGAAACC  reverse: TGAAGTCGCAGGAGACAACC |
| *IL-6* | forward: CCTAGTGCGTTATGCCTAAG  reverse: AGTGTCCCAACATTCATATTG |
| *RAGE* | forward: CGAGGGAAGGAGGTCAAGTC  reverse: GCCATCGGGAATCAGAAGT |
| *DDOST(AGER1)* | forward: CGCTGGGTGTTCAAGGAGGA  reverse: AGTGTAGGCATTGGGTGGAG |
| *Sirt1* | forward: AAGGAGCAGATTAGTAAGC  reverse: TAGAGGATAAGGCGTCAT |
| *Bax* | forward: TTTTGCTACAGGGTTTCATC  reverse: GTCCAGTTCATCTCCAATTC |
| *Bcl2* | forward: ACTTCTCTCGTCGCTACCGTC  reverse: AAGAGTTCCTCCACCACCGT |
| *P53* | forward: TACCTGAAGACCAAGAAG  reverse: ATAAGACAGCAAGGAGAG |
| *ZO-1* | forward: GATCGTCTGTCCTACCTGTC  reverse: CCGCCTTCTGTATCTGTGTC |

| **Table S4. Phytocomponents identified in the HE by GC-MS** | | | | | | |
| --- | --- | --- | --- | --- | --- | --- |
| **Peak no.** | **Compounds** | **RT** | **Quantity**  **(%)** | **MF** | **Biological activity^a^** | **Compound nature^b^** |
| **1** | 3-methyl-2-Hexanol, | 7.2816 | **0.115** | C7H16O | - | Fatty alcohol |
| **2** | Nitric acid, hexyl ester | 7.8951 | **4.153** | C6H13NO3 | Non-steroidal anti-inflammatory drugs (NSAIDs) | NSAID |
| **3** | Hydroperoxide, 1-methylhexyl | 9.2096 | **0.122** | C7H16O2 | - | - |
| **4** | 6-deoxy-3-C-methyl-2-O-methyl- L-Talose | 9.8814 | **0.02** | C8H16O5 | Anti-inflammatory | Methylglycosides |
| **5** | 2,2-dimethyl-3-methylene-bicyclo [2.2.1]heptane | 11.7121 | **0.188** | C10H16 | - | camphene |
| **6** | 2,4-Nonadiyne | 12.5349 | **0.465** | C9H12 | - | - |
| **7** | ethenyl-1,3,5-Trimethylbenzene OR 1,2,3-Trimethylbenzene | 12.9779 | **0.405** | C9H12 | - | mesitylene |
| **8** | Oxirane | 13.2944 | **0.657** | C4H6O | - | 3,4-epoxy-1-butene |
| **9** | 2,5-dihydro-Furan | 13.9711 | **0.105** | C4H8O2 | Anti-inflammatory | - |
| **10** | 2-methoxy-2H-Pyran-2-one, tetrahydro-6,6-dimethyl | 14.2584 | **0.919** | C7H12O2 | - | - |
| **11** | Phenol, | 15.0179 | **1.223** | C7H8O2 | - | Guaiacol |
| **12** | 1-(Cyclopropylazo)-1-spiropentanecarbonitrile | 15.6508 | **0.146** | C9H11N3 | - | - |
| **13** | 4-(hexyloxy)-1-Butanol | 15.7774 | **2.215** | C10H22O2 | Non-steroidal anti-inflammatory drugs (NSAIDs) | NSAID |
| **14** | 2-ethyl-1-Hexanol acetate | 16.0208 | **4.73** | C10H20O2 | - | 2-ethylhexyl acetate |
| **15** | 1,3-dihydroxy-2-methyl-Cycloheptano [d]imidazolidine, | 17.2769 | **0.036** | C7H7NO3 | Anti-inflammatory | benzyl alcohol |
| **16** | N-(aminoiminomethyl)-Acetamide | 17.5398 | **0.033** | C3H7N3O | - | - |
| **17** | Cycloheptatrienylium, iodide | 18.0364 | **0.301** |  | - | - |
| **18** | 2-nitro-Benzenemethanol | 18.4356 | **2.287** | C7H7NO3 | - | - |
| **19** | Dihydrocarvyl acetate | 18.8495 | **0.135** | C12H20O2 | - | - |
| **20** | 2-Nonynoic acid | 19.3558 | **0.113** | C9H14O2 | - | - |
| **21** | (E)-3-Hexen-1-ol, | 19.6236 | **0.022** | C6H12O | Anti-inflammatory | Fatty Alcohols-Hexanols |
| **22** | (Z,Z)-.alpha.-Farnesene | 20.0618 | **0.612** | C15H24 | Antioxidant, Anti-inflammatory, Antibacterial, Immunomodulator | Terpenes |
| **23** | (Z,E)-1,3,6,10-Dodecatetraene, 3,7,11-trimethyl-, | 20.1884 | **0.87** | C15H24 | Antioxidant, Anti-inflammatory, Antibacterial, Immunomodulator | Terpenes |
| **24** | 2,2-dimethyl-3,4-Pentadienal, | 20.349 | **0.378** | C7H10O | - | - |
| **25** | 1,1-Difluoro-2-methyl-3-ethyl cyclopropane | 20.6363 | **0.464** | C6H7F2 | - | - |
| **26** | Nerolidol | 21.0404 | **0.104** | C15H26O | Antioxidant, Anti-inflammatory, Antibacterial, Immunomodulator | Terpenes |
| **27** | N'-Hydroxypropimidamide | 21.1816 | **0.225** | C3H8N2O | - | - |
| **28** | 1,6-Bis(2-propyn-1-yloxy)hexane | 21.5759 | **0.157** |  | - | - |
| **29** | 17.alpha.-hydroxy-17.beta.-cyano-Preg-4-en-3-one, | 21.868 | **0.113** | C20H27NO2 | - | - |
| **30** | (E)-3-Hexen-1-ol, | 22.0871 | **0.039** | C6H12O | - | Fatty alcohol |
| **31** | 3-Methyl-2-butenoic acid, 3-phenyl-2-propenyl ester | 22.5253 | **0.4** | C14H16O2 | - | - |
| **32** | 4-(6-Oxabicyclo[3.1.0]hex-1-yl)-but-3-yn-2-one | 22.7444 | **0.727** | C9H10O2 | - | - |
| **33** | 2,2-dimethyl-3,4-Pentadienal, | 22.9537 | **0.273** | C7H10O | - | - |
| **34** | ethyl ester, Pentanoic acid | 23.4552 | **0.156** | C7H14O2 | Anti-inflammatory | Ethyl valerate |
| **35** | 17.alpha.-hydroxy-17.beta.-cyano-Preg-4-en-3-one, | 23.7522 | **0.23** | C20H27NO2 | - | - |
| **36** | (E,E)-3,5-Nonadien-7-yn-2-ol, | 24.1368 | **0.066** | C9H12O | - | - |
| **37** | 17.alpha.-hydroxy-17.beta.-cyano-Preg-4-en-3-one, | 24.3559 | **0.244** | C20H27NO2 | - | - |
| **38** | Propionic acid, 3-(2-methylcyclohexyl)-, ethyl ester | 25.5244 | **0.431** | C12H22O2 | Anti-inflammatory | Thiadiazines |
| **39** | Preg-4-en-3-one, 17.alpha.-hydroxy-17.beta.-cyano- | 26.0892 | **0.094** | C20H27NO2 | - | - |
| **40** | (Z)-3,6-Octadien-1-ol, 3,7-dimethyl-, | 26.2157 | **0.046** | C10H18O | Antibacterial, sedative | Isogeraniol |
| **41** | .alpha.-Humulene | 26.5273 | **1.252** | C15H24 | Antioxidant, Anti-inflammatory, Antibacterial, Immunomodulator | Terpenes |
| **42** | 2,6-Dimethyl-3,5,7-octatriene-2-ol, ,E,E- | 26.9509 | **0.045** | C10H16O | - | - |
| **43** | Preg-4-en-3-one, 17.alpha.-hydroxy-17.beta.-cyano- | 27.1067 | **0.184** | C20H27NO2 | - | - |
| **44** | 4,9-Decadienoic acid, 2-nitro-, ethyl ester | 27.4037 | **0.385** | C12H19NO4 | - | - |
| **45** | 7-Oxabicyclo[4.1.0]heptane, 1-methyl-4-(2-methyloxiranyl)- | 27.6471 | **11.247** | C10H16O2 | Anti-inflammatory, anti-obesity | Limonene dioxide |
| **46** | **(1S,2R,5E,9E,12R)-12-Isopropyl-1,5,9-trimethyl-15-oxabicyclo[10.2.1]pentadeca-5,9-dien-2-ol** | **27.8272** | **21.342** | **C20H34O2** | **Antioxidant, Anti-inflammatory, Antibacterial, Immunomodulator** | **Terpenes** |
| **47** | Estran-3-one, 17-(acetyloxy)-2-methyl-, (2.alpha.,5.alpha.,17.beta.)- | 28.1486 | **0.837** | C21H32O3 | - | - |
| **48** | 3-hydroxy-2-methyl-5-(prop-1-en-2-yl)cyclohexanone | 28.3239 | **1.193** | C10H16O2 | - | - |
| **49** | 4,8-Dioxatricyclo[5.1.0.0(3,5)]octane, 1-methyl-5 (1-methylethyl)-,(1.alpha.,3.alpha.,5.alpha.,7.alpha.)- | 28.4699 | **3.389** | C10H16O2 | Anti-inflammatory | cyclic ethers |
| **50** | (r-1,t-4)-8,9-epoxy-p-menthan-1-ol | 28.6744 | **5.683** | C10H18O2 | - | - |
| **51** | 8-(2-Acetyloxiran-2-yl)-6,6-dimethylocta-3,4-dien-2-one | 28.9032 | **1.59** | C14H20O3 | - | - |
| **52** | **Artemiseole** | **29.0493** | **8.854** | **C10H16O** | **Anti-inflammatory** | - |
| **53** | Estran-3-one, 17-(acetyloxy)-2-methyl-, (2.alpha.,5.alpha.,17.beta.)- | 29.1905 | **6.43** | C21H32O3 | - | - |
| **54** | 2-Nitro-2-(3-oxobutyl) cycloheptanone | 29.614 | **0.425** | C11H17NO4 | - | - |
| **55** | 2,3-Dioxabicyclo[2.2.1]heptane, 1-methyl- | 30.0327 | **1.243** | C6H10O2 | - | - |
| **56** | 6-(3-Methyl-3-cyclohexenyl)-2-methyl-2,6-heptadienol | 30.2275 | **2.749** | C15H24O | - | - |
| **57** | 9,12,15-Octadecatrienoic acid, 2,3-bis(acetyloxy)propyl ester, (Z,Z,Z)- | 30.6218 | **1.082** | C25H40O6 | - | - |
| **58** | 4,8-Dioxatricyclo[5.1.0.0(3,5)]octane, 1-methyl-5-(1-methylethyl)-,(1.alpha.,3.alpha.,5.alpha.,7.alpha.)- | 30.7874 | **4.527** | C10H16O2 | Anti-inflammatory | cyclic ethers |
| **59** | Androstan-3-one, 17-hydroxy-2-methyl-, (2.beta.,5.beta.,17.beta.)- | 31.0503 | **0.674** | C20H32O2 | - | - |
| **60** | 4,8-Dioxatricyclo[5.1.0.0(3,5)]octane, 1-methyl-5-(1-methylethyl)-,(1.alpha.,3.alpha.,5.alpha.,7.alpha.)- | 31.318 | **0.938** | C10H16O2 | - | - |
| **61** | .alpha.-Citronellyl acetate, 6-chloro- | 32.4524 | **0.284** | C12H21Cl2 | - | - |
| **62** | Isopulegol | 32.691 | **0.18** | C10H18O | Antioxidant, Anti-inflammatory, Antibacterial, Immunomodulator | Terpenes |
| **63** | Butane, 1,2-dibromo- | 33.543 | **0.433** | C4H8Br2 | Sedative, Antidepressant | Opioids |
| **64** | Diazoprogesterone | 37.8177 | **0.664** | C21H30N4 | Anti-inflammatory, anti-colitis | Progesterone |
| HE: Herbal extract; RT: Retention time; MF: Molecular Formula. ^a^ Biological Activity Source: Dr. Duke's Phytochemical and Ethnobotanical Database; ^b^ Compound Nature Source : pubchem.ncbi.nlm.nih.gov/compound. Most abundant components are bolded and underlined. | | | | | | |

**Supplementary Figs:**


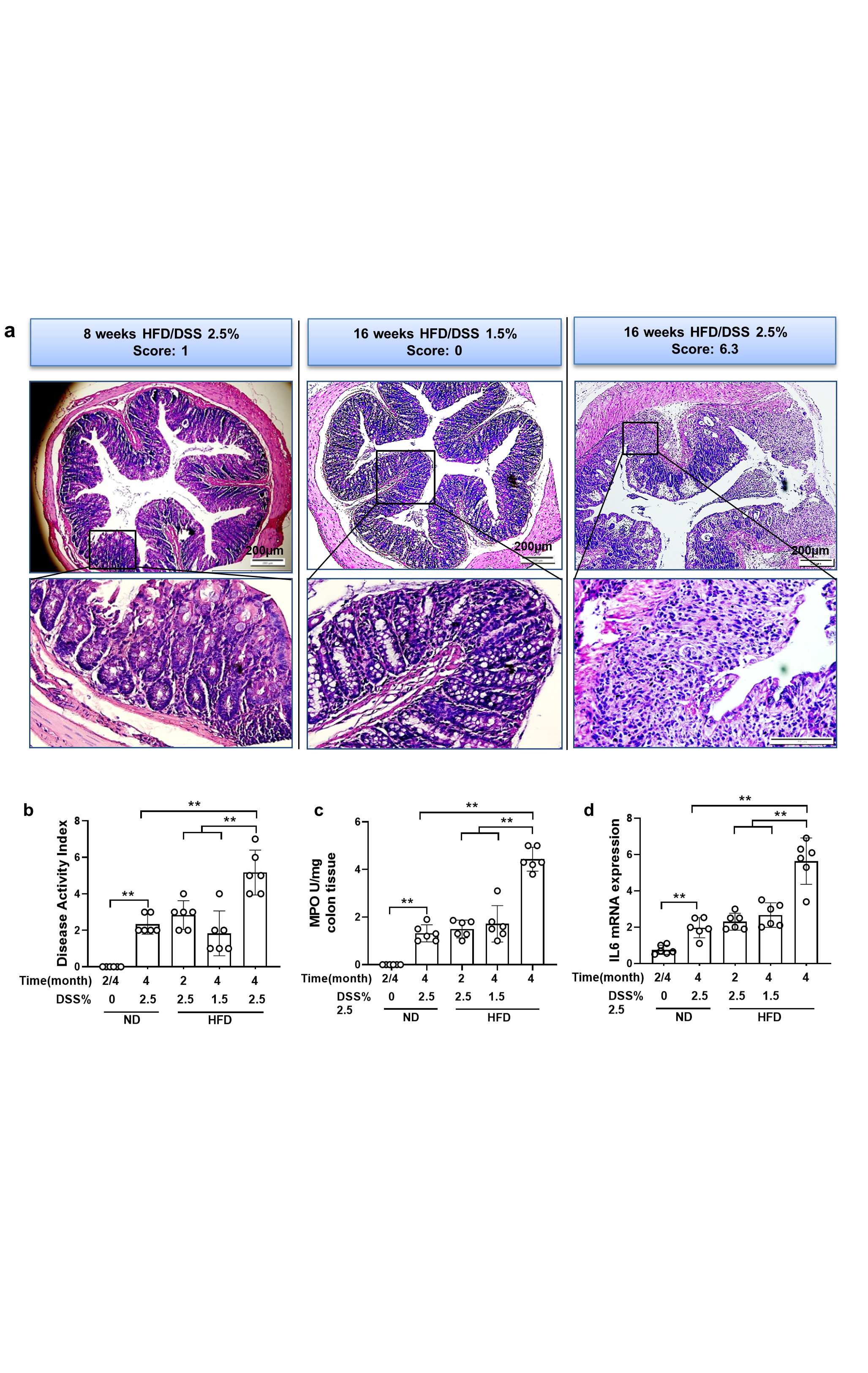


**Figure S1.** **Determination of optimal concentration of DSS dose and diet duration.** (**a**) Representative images of hematoxylin and eosin (H & E) staining of distal colonic sections (at least 5 parts were evaluated for each sample), (**b**) Disease activity index, (**c**) MPO activity, (**d**) mRNA expression of pro-inflammatory cytokine IL-6. ND: normal diet; HFD: high AGEs-fat diet; DSS: 2.5% dextran sulphate sodium; MPO, Myeloperoxidase. Data as mean with 95% CI, n=6 per group. * and ** respectively represents p<0.05 and p<0.01.


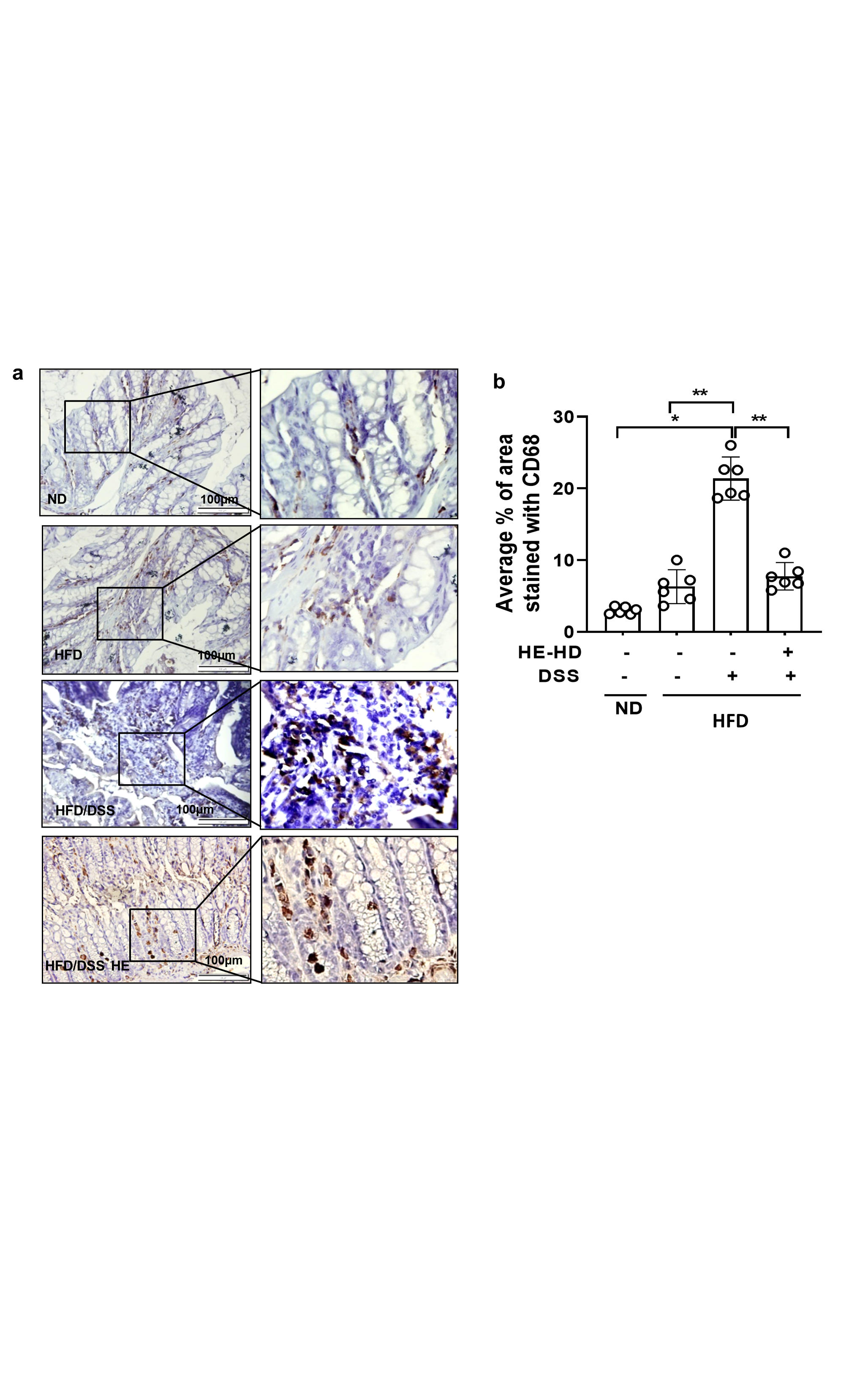


**Figure S2. Immunohistochemical staining of macrophages in colon.** (**a**) Representative staining shown for CD68 (at least 5 parts were evaluated for each sample). (**b**) Quantitative analysis of CD68 IHC staining. HE: herbal extract; ND: normal diet; HFD: high AGEs-fat diet; HD: high dose; LD: low dose; MD: mid dose; DSS: 2.5% dextran sulphate sodium. Data as mean with 95% CI, n=6 per group. * and ** respectively represents *p*<0.05 and *p*<0.01.


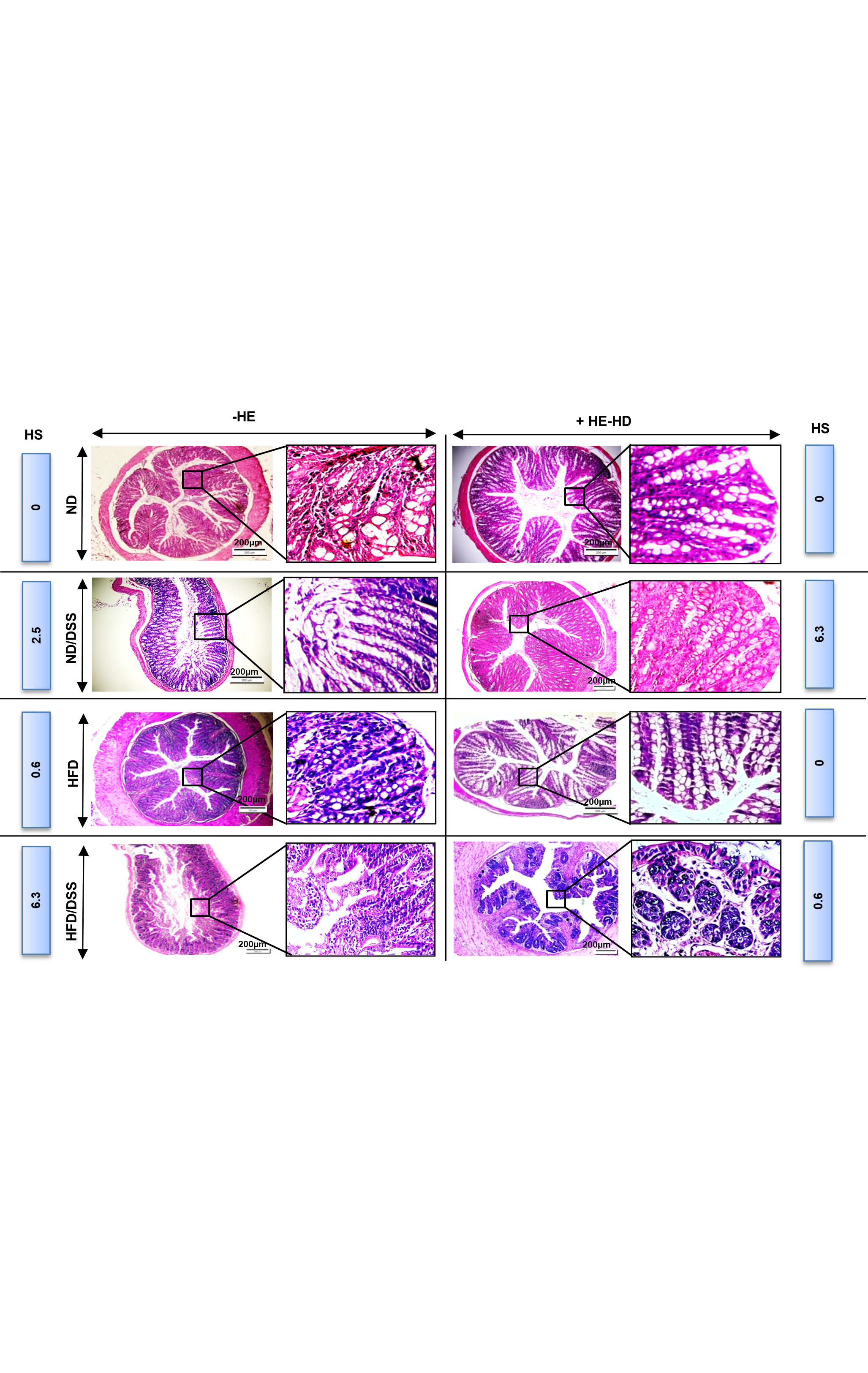


**Figure** S**3. Representative images of H&E staining of distal colonic sections.** HE: herbal extract; ND: normal diet; HFD: high AGEs-fat diet; HD: high dose; DSS: 2.5% dextran sulphate sodium. HS: Histological score. Data as mean with 95% CI, n=6 per group, at least 5 parts were evaluated for each sample.


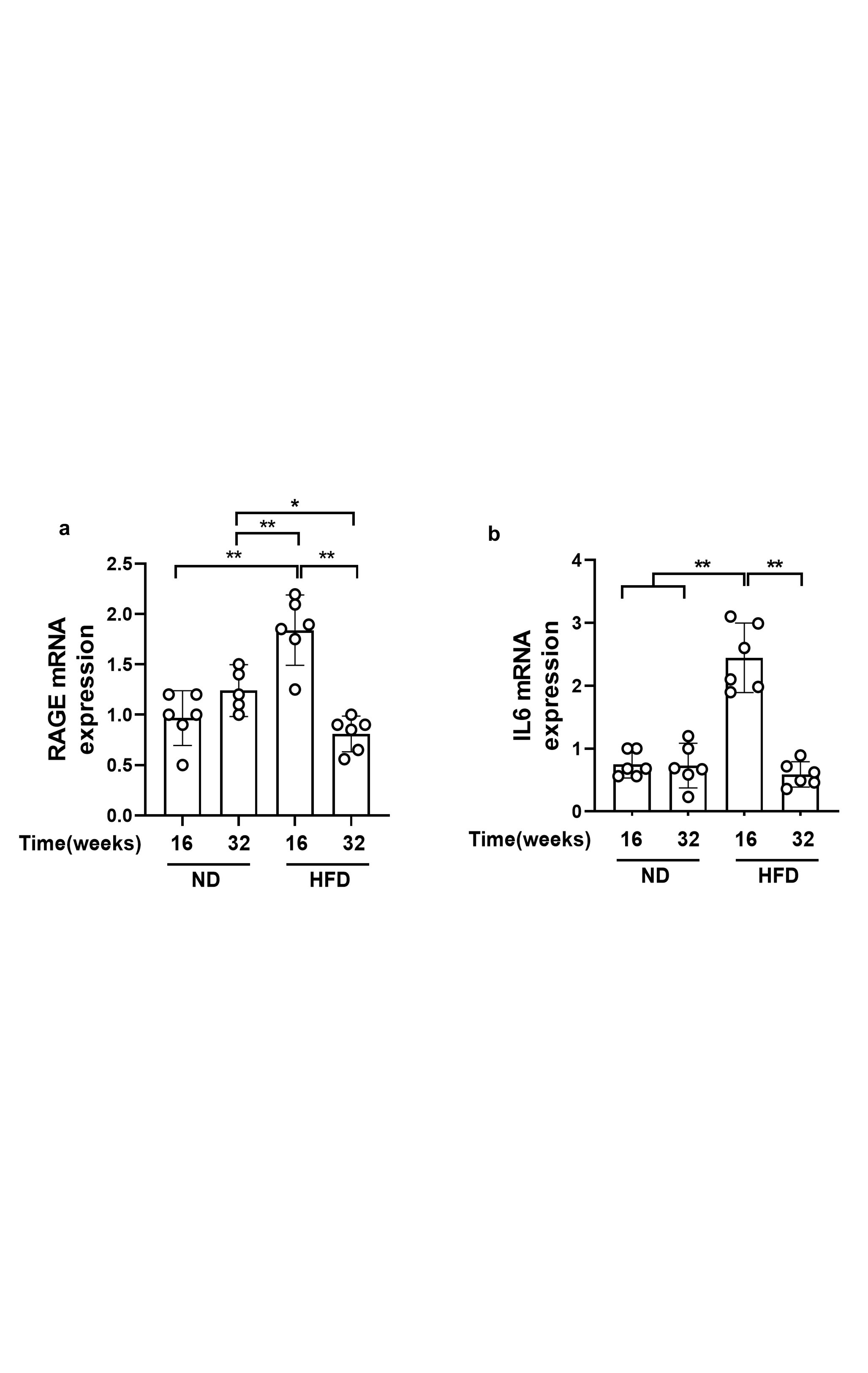


**Figure S4. Comparison of 4 and 8 months HFD consumption.** mRNA expression of (**a**) RAGE and (**b**) IL-6. ND: normal diet; HFD: high AGEs-fat diet; DSS: 2.5% dextran sulphate sodium. Data as mean with 95% CI, n=6 per group. * and ** respectively represents p<0.05 and p<0.01.
